# Care of older people with Cognitive Impairment or Dementia Hospitalized in Traumatology Units (CARExDEM): a quasi-experiment

**DOI:** 10.1186/s12877-020-01633-5

**Published:** 2020-07-16

**Authors:** Claudia Casafont, Ester Risco, Mercè Piazuelo, Marta Ancín-Pagoto, José Luis Cobo-Sánchez, Montserrat Solís-Muñoz, Adelaida Zabalegui

**Affiliations:** 1 Consultant Nurse in Research, Hospital Clinic Barcelona, Assistant lecturer Universitat de Barcelona, Villarroel, 170, 08036 Barcelona, Spain; 2 Associate Nursing Director. Hospital d’Atenció Intermèdia Parc Sanitari Pere Virgili, Esteve Terrades,30, 08023 Barcelona, Spain; 3 Nurse Unit Manager in Traumatology Unit, Hospital Clinic Barcelona, Villarroel, 170, 08036 Barcelona, Spain; 4 Vice director of Nursing Care in Complejo Hospitalario de Navarra, Pabellón G. Irunlarrea,3, 31008 Pamplona, Spain; 5 Consultant Nurse in Research, Hospital Universitario Marqués de Valdecilla, Santander. Pabellón 16 Planta baja. Avenida Valdecilla s/n., 39008 Santander, Spain; 6 Head of the Care Research Unit, Puerta de Hierro Majadahonda University Hospital. Head of the Nursing and Health Care Research Group, Puerta de Hierro-Segovia de Arana Health Research Institute, Joaquín Rodrigo, 2, 28222 Madrid, Majadahonda Spain; 7 Vice director of Research and Education in Nursing in Hospital Clinic Barcelona, Assistant lecturer Universitat de Barcelona, Escala 1 planta 7. Villarroel 170, 08036 Barcelona, Spain

**Keywords:** Dementia, Intervention, Elderly, Nursing care, Femur fracture, Continuity of patient care

## Abstract

**Background:**

In our context, as in other European countries, care of patients with cognitive disorders or dementia still represents a major challenge in hospital settings. Thus, there is a need to ensure quality and continuity of care, avoiding preventable readmissions, which involve an increase in public expenses. Healthcare professionals need to acquire the necessary knowledge and skills to care for hospitalized patients with cognitive disorders and dementia.

**Methods:**

A quasi-experimental design with repeated observations, taken at baseline, post-intervention, and at one and three months post-intervention, in people hospitalized with cognitive disorders or dementia. The study will be carried out in four general hospitals in Spain and will include 430 PwD and their caregivers. The intervention was previously developed using the Balance of Care methodology where nurses, physicians, social workers and informal caregivers identified the best practices for this specific care situation. We aim to personalize the intervention, as recommended in the literature. The study has an innovative approach that includes new technologies and previous scientific evidence. Valid, reliable instruments will be used to measure the intervention outcomes. Quality of care and comorbidity will be analyzed based on the use of restraints and psychotropic medication, pain control, falls, functional capacity and days of hospitalization. Continuity of care will be measured based on post-discharge emergency hospital visits, visits to specialists, cost, and inter-sectorial communication among healthcare professionals and informal caregivers. Statistical analysis will be performed to analyze the effect of the intervention on quality of care, comorbidity and continuity of care for patients with dementia.

**Discussion:**

Our aim is to helping healthcare professionals to improve the management of cognitive disorders or dementia care during hospitalization and the quality of care, comorbidity and continuity of care in patients with dementia and their informal caregivers. Moving towards dementia-friendly environments is vital to achieving the optimum care outcomes.

**Trial registration:**

Registered in Clinical Trials. ClinicalTrials.gov Identifier: NCT04048980 retrospectively registered on the 6th August 2019. https://clinicaltrials.gov/

Protocol Record HCB/2017/0499.

Sponsor: Hospital Clinic Barcelona.

## Background

Acute hospital care of patients with dementia (PwD) needs to be redesigned so that it is tailored to their needs and those of their caregivers. This would allow improvements to care received and continuity of care, along with a reduction in costs.

Fifty million people worldwide live with dementia. Dementia has still no cure and is linked to aging [1]. According to the National Statistics Institute in Spain (INE), it is expected that 29.4% of the total population will be 65 or older in 2068 [2]. As dementia progresses, cognitive and functional levels deteriorate until PwD depend completely on a caregiver [3]. An informal caregiver (IC) is a person who assists or looks after another person affected by an impairment or disability that prevents or impedes normal performance of daily-life activities or social relationships without receiving any income. Usually, ICs are women within the family between 45 and 70 years old [4]. Informal care costs in Spain represent a growing economic burden [5], higher than formal care costs.

Dementia is one of the main causes of incapacity in the elderly, requiring frequent health and social service use. In recent years, the number of hospitalized patients with dementia has increased, although 25% of these patients are not formerly diagnosed [6]. For this reason we classify included patients as those with dementia or cognitive impairment.

In Spain, about 50,000 to 60,000 femur fractures occur every year, with a hospitalization rate of 100 admissions per 100,000 inhabitants/year [7]. A study showed that 38.5% of patients older than 70 admitted due to a femur fracture were PwD [8], similar to findings by Mosk’s et al. [9]. Hence, we focus on this population group, due to the high incidence of dementia and femur fractures seen in the Spanish National Health System.

When a PwD is admitted to a fast-paced, noisy acute hospital, the unknown environment can trigger disorientation and agitation, where distress is also experienced due to disruption of normal routine [10]. This can be challenging for nurses that are expert in the unit specialty, in this case traumatology, but are not specifically trained to look after PwD. Lack of training focused on care of PwD or cognitive impairment can cause professionals to feel incompetent and frustrated and lead to high levels of stress. This can result in heightened clinical practice risk [11, 12]. Additionally, the unpredictable nature of dementia means that PwD can show lucidity one day, or inability to follow instructions the next day, which can worsen the situation [13]. As such, educational interventions should focus on how staff identifies PwD experiences and shares knowledge on how to manage challenging behaviors through development of person-centered practices and focusing on priorities [14].

A study in the United Kingdom showed that 57% of hospitalized PwD presented aggressiveness, 42% had sleeping problems and 35% suffered from anxiety [15]. To prevent delirium and aggressiveness in PwD, we can act on triggers, modify the environment, foster psychosocial abilities or optimize resources. Furthermore, physical restraints are used to control agitation/aggressiveness when there is potential danger to the patient and his/her environment and he/she cannot be controlled with other measures (verbal or pharmacological). Restraints can only be used when other measures fail [16]. However, in Spain, 25% of hospitalized patients with moderate to severe dementia presenting agitation are physically restrained; one of the highest rates in countries in Europe [17].

Similarly, psychotropic medication administration is also higher in Spain than in any other European country, identified in 54% of PwD [18]. Studies found an association between use of multiple psychotropic medications and a higher risk of fall injuries, hospitalizations and death. Non-pharmacological alternatives are safer and can impact positively in social economic burden and patient comfort [19].

Another important aspect to consider in care of hospitalized PwD is pain management. This can also be very challenging for nurses, especially when it comes to initiating pain assessment and adequate use of tools. Moreover, cognitive impairment limits the ability to communicate and describe pain [20].

Very few intervention studies are found in the literature and more research needs to be conducted to develop and assess educational programs to improve care of hospitalized PwD. Moreover, communication skills between multidisciplinary and intersectoral teams and patients and families need to be improved to ensure better global health services [21]. Furthermore, health professionals looking after PwD should have specific skills and knowledge to provide adequate care [22]. PwD are still stigmatized, and nurses should understand their needs and be empathic as with any other patients [10]. For example, training staff in nursing homes through didactic sessions and case discussions given by experts in neurology and geriatrics decreased the use of physical and chemical restrictions [23]. The same could be applied in hospital settings. The high vulnerability of hospitalized PwD underlines the need to confront this challenge continuously from a global perspective and the importance of conducting intervention studies in hospitalized PwD at the national and international level. Therefore, we propose to design, implement and assess a multidisciplinary, multifactorial educational intervention called “CARExDEM”, based on the Balance of Care model (BoC) [24], addressed to nurses looking after hospitalized PwD in traumatology units.

The outlined protocol follows the research group approach to care of PwD, highlighting the following studies: 1) RightTimePlaceCare for PwD and their caregivers (RTPC), financed by the EU (grant agreement 242,153) [17]. This study compared care of PwD in eight countries to identify best practices related to quality of care and quality of life of PwD and their carers. 2) The study “Information, training and Social support” (INFOSA) [25], for caregivers of dependent, elderly patients admitted to hospitals, financed by FIS grant (PI 09/00111) and 3) Experimental study INFOSA-DEM for caregivers of PwD financed by “La Marató de la TV3” Exp. 20,144,410 (Reference pending for publication in JAN). We also draw on the experience and knowledge generated by the European study on empowerment of patients admitted to traumatology units for surgery [26] that demonstrates how greater satisfaction with received care promotes better postsurgical recovery.

We expect to decrease comorbidity and improve quality and continuity of care of hospitalized PwD with proximal femur fracture requiring surgery, along with minimizing costs.

## Aims and objectives

The aim of this study is to design, implement and assess the effectiveness of an intervention program for nurses looking after hospitalized patients with proximal femur fracture and dementia or cognitive impairment in acute hospitals and measure its impact in terms of quality of care, morbidity reduction, improvement of continuity of care, and cost reduction. It has 4 objectives:

1. To develop and implement a program for care of hospitalized patients with dementia or cognitive impairment (CARExDEM) with a multidisciplinary consensus model (Balance of Care).
2. To assess the impact of the intervention regarding quality of care and comorbidity in these patients (physical restraints, psychotropic medication, falls, functional capacity, pain assessment, length of hospital stay and number of readmissions).
3. To evaluate the impact of the (CARExDEM) intervention with respect to to continuity of care (informal caregiver reaction, multidisciplinary and intersectoral communication) in global patient care.
4. To assess the impact of the intervention CARE x DEM regarding the economic costs of hospital and community care at 1 and 3 months post discharge.
5. To evaluate nurses’ knowledge before and after implementing CARExDEM.

## Methods

### Design

**Fig. 1:**
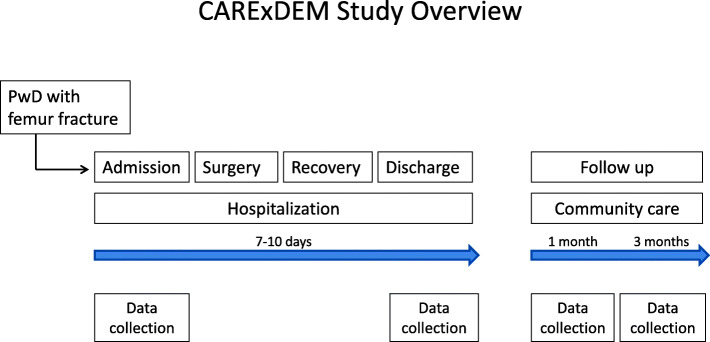
Study Protocol Overview

This is a quasi-experimental pre-post test, longitudinal, multicenter study. Data will be collected at hospital admission, discharge, 1 month follow-up (when visiting physician for surgery follow-up) and 3 month follow-up (See Fig. 1 for study overview).

### Setting and participants

The study will be conducted in four traumatology units at high technology public hospitals across Spain. The autonomous communities included are Catalonia (Hospital Clinic Barcelona), Madrid (Hospital Puerta de Hierro Majadahonda), Cantabria (Hospital Universitario Marqués de Valdecilla) and Navarra (Complejo Hospitalario de Navarra). The study will be held in a 3-year frame time, with control group data collection starting in in August 2018.

Participants will be PwD and their caregivers (*n* = 432), and healthcare professionals working in the traumatology unit (*n* = 85). Patients will be recruited consecutively in two groups from emergency department admissions for surgery (See Table 1):

- Control group: People with dementia or cognitive impairment hospitalized with proximal femur fracture under surgery and their informal caregivers receiving usual nursing care. The main aspects covered in usual nursing care include control of pain, mobility, drainage and wound assessment, mobilization and nutrition following the established guidelines for each hospital.
- Experimental group: People with dementia or cognitive impairment hospitalized with proximal femur fracture under surgery and their informal caregivers receiving the intervention (CARExDEM).
- All healthcare professionals working in traumatology units (nurses, care assistants, physiotherapists, social workers and physicians) will answer an ad hoc questionnaire to assess knowledge in care of PwD before and after the intervention.

**Table 1:** Data collection summary. (*HCB* Hospital Clinic Barcelona, *HPHM* Hospital Puerta Hierro Majadahonda, *HMV* Hospital Universitario Marqués de Valdecilla; *CHN* Complejo Hospitalario de Navarra)

	Data collection 1	Data collection 2	Data collection 3	Data collection 4
**Control Group** People with dementia or cognitive impairment admitted to traumatology units receiving usual care	HCB (*n* = 54)	HCB (*n* = 54)	HCB (*n* = 54)	HCB (*n* = 54)
	HPHM (*n* = 54)	HPHM (*n* = 54)	HPHM (*n* = 54)	HPHM (*n* = 54)
	HMV (*n* = 54)	HMV (*n* = 54)	HMV (*n* = 54)	HMV (*n* = 54)
	CHN (*n* = 54)	CHN (*n* = 54)	CHN (*n* = 54)	CHN (*n* = 54)
INTERVENTION: CARExDEM Program				
**Experimental Group** People with dementia or cognitive impairment admitted to traumatology units receiving the intervention	HCB (*n* = 54)	HCB (*n* = 54)	HCB (*n* = 54)	HCB (*n* = 54)
	HPHM (*n* = 54)	HPHM (*n* = 54)	HPHM (*n* = 54)	HPHM (*n* = 54)
	HMV (*n* = 54)	HMV (*n* = 54)	HMV (*n* = 54)	HMV (*n* = 54)
	CHN (*n* = 54)	CHN (*n* = 54)	CHN (*n* = 54)	CHN (*n* = 54)

Inclusion criteria:

- Patients older than 65 hospitalized for surgery; a score of 5 or less in the Short Portable Mental Status Questionnaire (SPMSQ) test [27]; providing signed informed consent; and with an informal caregiver able to understand the recommendations of health professionals.
- Informal caregivers: The person in charge of looking after the patient with dementia, living with him/her or visiting at least three times per week at home or at a nursing home; providing signed informed consent.

Exclusion criteria: Patients younger than 65; those with psychiatric symptoms or Korsakoff’s syndrome, absence of signed informed consent.

## Measurements

### Measures for PwD

All questionnaires selected are valid, reliable and widely used among studies with PwD. All questionnaires had previously been translated into Spanish and authors gave permission for their use. The whole questionnaire can be completed in 45 min (for patient questions) and 15 min (for informal caregivers). Table 2 represents a summary of all tests included and their main features.

**Table 2:** Distribution of questionnaires for all data collection phases

Questionnaires	Number of Items	Admission	Discharge	1-month follow-up	3-month follow-up
**PwD and IC**					
Inclusion and exclusion criteria	–	✓			
Cognitive Assessment (SPMSQ)	10	✓	✓		
Clinical data and history	–	✓			
Medication record	–	✓	✓	✓	✓
PAINAD	5	✓	✓	✓	✓
Morbidity (falls, pressure ulcers, restraints, etc.)	–	✓	✓	✓	✓
Nutritional assessment (MNA)	6 + 12	✓		✓	
Charlson Comorbidity index	19	✓			
Barthel	10	✓	✓	✓	✓
ADL (Katz)	6	✓	✓	✓	✓
NPI-Q	12	✓	✓	✓	
Satisfaction (PSS)	11		✓		
Costs (RUD)	–	✓		✓	
Caregiver Reaction (CRA)	24			✓	
Caregiver experience (IEXPAC)	16	✓	✓	✓	✓
Follow –up (use of health and social services)	–			✓	✓

Measurements performed in PwD include use of mechanical restraints, pain management, number of falls, comorbidity (Charlson Index) [28, 29], nutritional assessment (MNA) [30, 31], pressure ulcers, psychotropic medication administration, functional capacity (Barthel) [32, 33], length of stay and readmissions, behavior (NPI) [34, 35], activities of daily living (Katz) [36, 37], and pain assessment for PwD (PAINAD) [38, 39].

Continuity of care: Nursing discharge planning and information given to patients and families.

### Measures for informal caregivers

Patient satisfaction with nursing care (PSS) [40].

Care reaction (CRA) [41, 42] measuring self-esteem, lack of family support, financial problems, interrupted schedules and health problems.

Continuity of care experienced from the IC’s perspective (IEXPAC) [43].

Costs: Resource utilization in dementia (RUD), to assess costs of formal, informal and community care in PwD [44].

### Measures for healthcare professionals

An ad hoc questionnaire will assess nurses’ and other healthcare professionals’ standards of care in PwD along with self-assessment on patient education prior to and following the intervention. Some open questions will identify barriers and facilitators in patient education and care.

## Data collection

Data will be collected by trained interviewers at hospital admission (within 24 h), on discharge, 1 month follow-up at the outpatient traumatology appointment and 3-month follow-up (phone call). Questionnaires have been standardized in one document according to the collection phase. Questions will be responded to according to patient and caregiver data.

## Procedure

The study will be conducted in 3 phases:

### Pre-intervention stage: program design and control group data collection

#### Intervention

Design of the CARExDEM intervention is based on results obtained from the European RightTimePlaceCare [17] study, where care of PwD and their caregivers was assessed in Europe using the Balance of Care methodology [24].

The research team organized two expert-panel meetings, inviting 20 healthcare professionals (nurses, physicians, physiotherapists, social workers and care assistants) with expertise in dementia, cognitive impairment and traumatology in hospitalization and primary care. They were divided into four groups and the research team provided each group with two clinical situations and a list of available resources and activities. The aim was to match the best resources to each situation to achieve the best care. The experts reached a consensus on the best care needed under each set of circumstances in relation to applicability, follow-up, population participation and cost/time saving. Results were validated in a second meeting with experts where activities were classified into five categories: study of basic care, organization, cognition, knowledge, safety, along with discharge planning. Intervention activities are included in Table 3.

**Table 3:** Intervention activities

Basic Care	PAINAD scale (pain assessment) [38, 39], Braden scale (pressure ulcer assessment) [45], skin integrity assessment, nutrition assessment [31], favoring night sleep (lights, noise, temperature…), minimizing physical restraints with verbal containments, negotiating and using TOP5 tool [46]; a strategy to enhance communication and enhancing patient-centered care.
Organization	Room placed next to nurses’ station, visible signs for toilet and wardrobe, staff identification badges.
Cognition	Patients wearing own gowns/clothes, memory exercises, pleasant reminiscence objects (photographs, music…). Visible clocks and calendars for time orientation. Volunteers will visit at least twice a week.
Knowledge	Brochure will be developed for caregivers and families to provide hospitalization recommendations and discharge planning.
Safety	Falls risk and cognitive impairment identification, closed slippers, physiotherapy aids, placing reachable objects, bed in low position, consider handrails and reachable bell.
Discharge planning	Early discharge planning, social worker follow-up, community resources information, contact with primary nurse.

Data will be collected for control group by trained interviewers. Subjects will be recruited by consecutive sampling on the first 24 h of ward admission.

### Implementation of the CARExDEM intervention

Implementation will follow the Promoting Action on Research Implementation in Health Services (PARIHS) framework [47], taking into account the three key elements to achieve successful implementation; evidence, context and facilitation.

Prior to implementation, educational training will be given, following the same protocol in each hospital. Training will be provided on aspects such as use of physical restraints, psychotropic medication, falls, functional capacity and pain management according to best practices and clinical guidelines. To enhance adherence and raise awareness about the care model, the intervention will be supported with a checklist. This will summarize all activities to be delivered to the patient by category; basic care, organization, cognition; knowledge, safety and discharge planning. It will be completed every 3 days from admission by the day shift nurse responsible for each patient.

A pilot test will be conducted prior to implementation in each hospital. We will consider the opinions of experts and informal caregivers related to applicability, follow-up, population participation, and time/cost savings. Once the intervention is implemented, the research team will monitor closely to be aware of questions in case of doubts or barriers. Weekly meetings will be held to review nurses’ training and to share their experiences with the research team.

### Follow-up and assessment of the CARExDEM implementation

After implementation, data will be collected for the experimental group using the same instruments and organization as those for the control group. Weekly meetings will be held for follow-up and to provide support to the interviewers and ward staff. A data manager will input data into a database and results will be evaluated by an expert statistician. Results will be disseminated through journals and congresses.

## Statistical analysis

Descriptive analysis will be used for baseline data. Categorical variables will be represented with absolute frequencies and percentages. For continuous variables, mean values, standard deviation or medians will be calculated. To study the effect of the intervention, analysis of co-variance will be carried out (ANCOVA), with Student’s t-test for continuous variables and McNemar’s test for categorical variables. Sensitivity will be analyzed to compare basal data of participants who complete the study with those who do not. Main outcome measures include quality of care and comorbidity (physical restraints, psychotropic medication, falls, functional capacity, pain assessment, length of hospital stay and number of readmissions); continuity of care (informal caregiver reaction, multidisciplinary and intersectoral communication) economic costs of hospital and community care and nurses’ level of knowledge in acute care of PwD. Outcomes measured will assess longitudinal changes from all the collection phases; baseline (admission), discharge, 1 month and 3-month follow-up, comparing pre-intervention and post-intervention values. Confidence intervals of 95% will be calculated. Values of *p* < 0.05 will be considered significant. Statistical analysis will be performed with R-3.2.3. for Windows. The research team will reflect on the analyzed data and issue a final report including the CARExDEM program with the required modifications identified following the implementation evaluation. This will ease the subsequent implementation of the program in other hospitals.

For the sample calculation, we estimated that a sample of 432 participants need to be included in the study, assuming an improvement in patients with pain around 50%, with precision of 5%, a confidence interval of 95 and 10% of dropouts. Only the research team and data manager will have access to data.

## Study progress

Data collection for the control group started in July 2018 and is ongoing. So far, 173 patients have been recruited in control group. Multidisciplinary groups are currently working on the intervention at each hospital, following the BoC methodology.

## Discussion

This paper presents the study protocol for the CARExDEM program. This intervention aims to provide nurses with strategies for the management of PwD in acute care and to raise awareness of the importance of individualized care in these patients to improve continuity of care in exacerbated situations in PwD. Hospitals in Spain are still far from being environments prepared for PwD. Most focus on quick and efficient diagnosis and management of acute serious disease processes, whereas other countries are now restructuring their units to be more senior friendly.

The presentation of the comprehensive CARExDEM care management protocol for PwD is innovative as we have found no published interventions for these patients in hospital settings. There is a need to ensure continuity of care so patients can return to their setting with minimal disruption. Informal caregivers will also benefit from a smoother transition after hospital discharge.

The main limitation of the study is its scope of application. As it is a study carried out in specialized units, the results obtained cannot be generalized to the other wards in the hospital. To compensate this limitation, we have included 4 hospitals from different Autonomous Communities to broaden project development in different clinical settings. A strength of this study is the information that contributes to empowering patients, their caregivers and professionals and encourages policy-makers and organizations to adapt hospital settings to the needs of PwD.

## Data Availability

The data that support the findings of this study are available on request from the corresponding author, (AZ).

## Abbreviations

ADL: Activity of daily living
BoC: Balance of Care
CARExDEM: Care of persons with cognitive impairment or dementia hospitalized in acute hospitals
CHN: Complejo Hospitalario de Navarra
CRA: Caregiver reaction assessment
IC: Informal caregiver
IEXPAC: Instrument for evaluation of experience of Chronic Patients
HCB: Hospital Clinic Barcelona
HPH: Hospital Puerta Hierro Madrid
HMV: Hospital Marqués de Valdecilla
MNA: Mini-nutritional assessment
NPI-Q: Neuropsychiatric Inventory Questionnaire
OCDE: Organization for Economic Cooperation and Development
PARIHS: Promoting Action on Research Implementation in Health Services
PAINAD: Pain assessment in advanced dementia
PwD: Patient with dementia
PSS: Patient Satisfaction Scale
RTPC: RightTimePlaceCare Study
SPMSQ: Short Portable Mental Status Questionnaire
RUD: Resource utilization in dementia
